# Supplementary material for: Ti3C2 MXene Membranes for Gas Separation: Influence of Heat Treatment Conditions on D-Spacing and Surface Functionalization
Source: Membranes (Basel). 2022 Oct 21;12(10):1025. doi: 10.3390/membranes12101025 (PMC9608636; doi:10.3390/membranes12101025)
Supplement: Supplementary file 1 [file membranes-12-01025-s001.zip › membranes-1967090-supplementary.pdf]

membranes

MDPI

Supplementary Materials

Ti_3C_2 MXene membranes for gas separation: influence of heat treatment conditions on d-spacing and surface functionalization

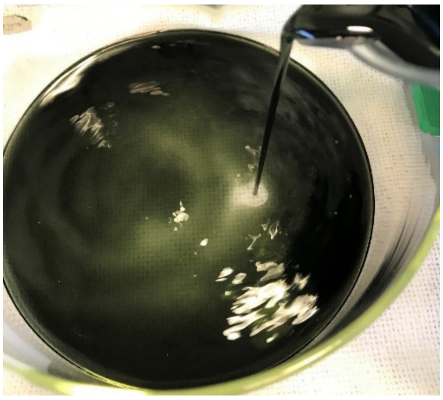

Figure S1. Dark greenish supernatant after the third cycle of centrifugation.

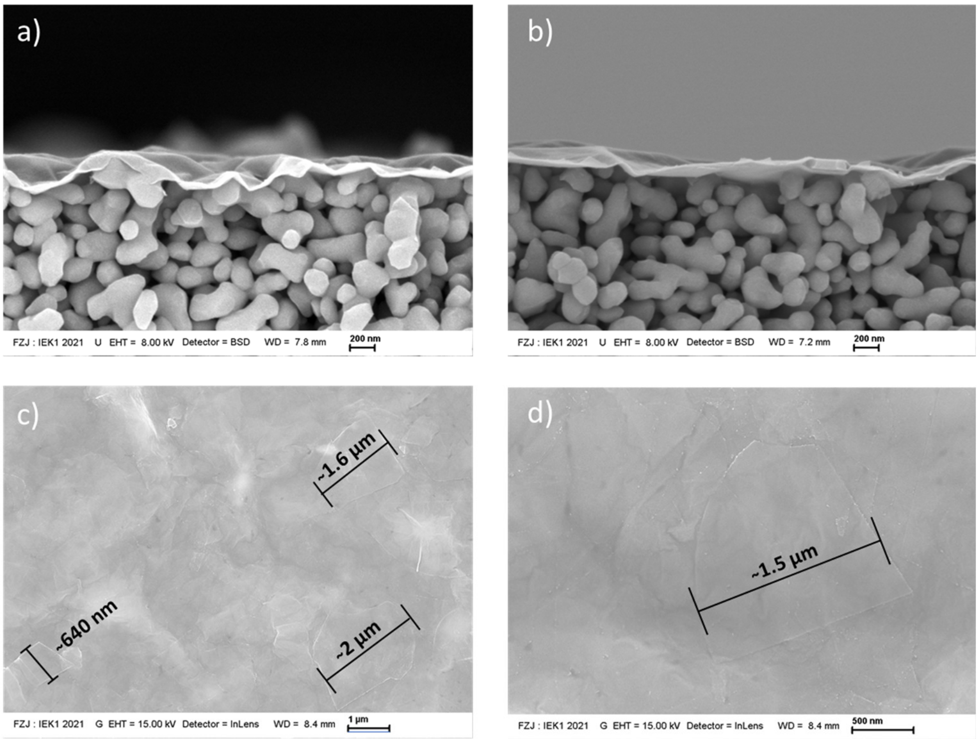

Figure S2. α-Al_2O_3 porous substrates coated with nano dispersion of $Ti_2C_3T_x$ flakes. Pore size of substrate presented an approximately value exceeding 200 nm. Surface morphology showing a few sizes of MXene flakes which indicates different sizes, from 640 nm to 2 μm, as

membranes MDPI

can be seen in panel (a), which shows a membrane ~20 nm-thick and panel (b), showing a ~40 nm-thick membrane.

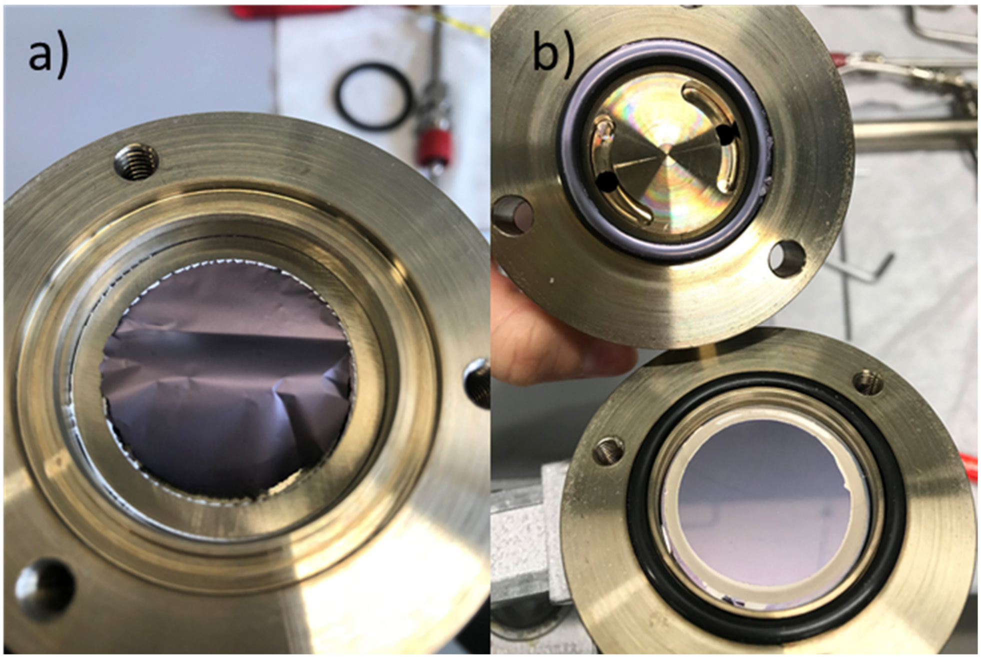

Figure S3. Free standing membrane after the assembling inside the metal massive module (a) and Thin film dip coated on alumina oxide substrate after assembling into de module (b). In the first case it was not possible the single gas permeation test through the free-standing membrane. In the second case, the thinner membrane attached to the hard substrate could capable to deal with the assembling and permeation tests.

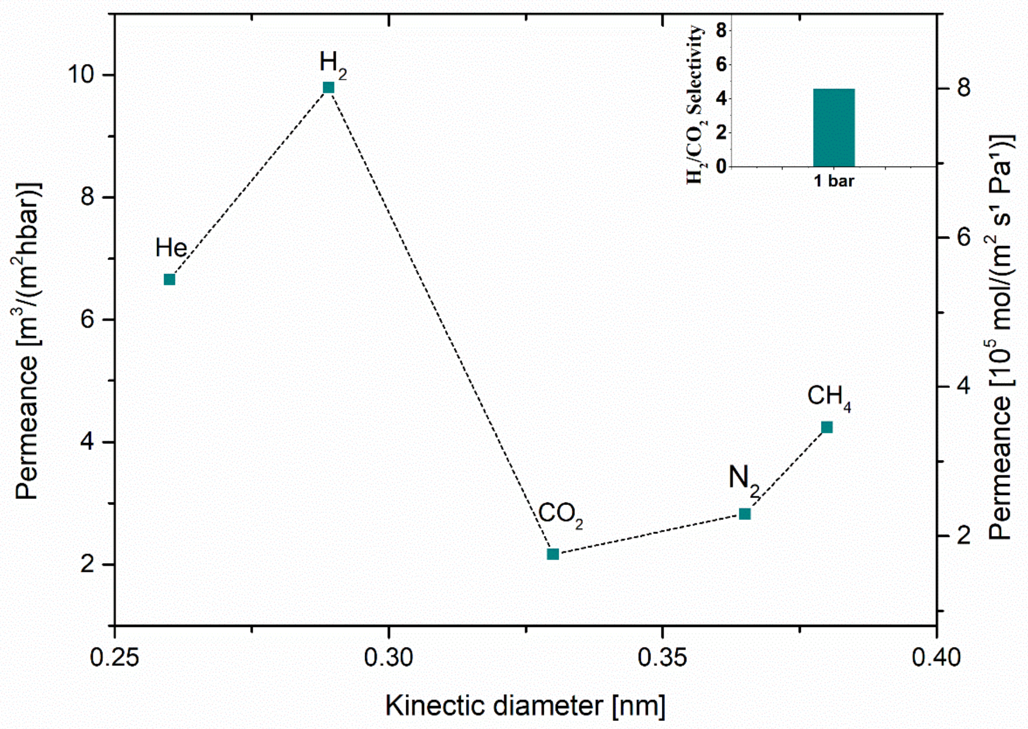

Figure S4. Single gas permeation through the substrate at 1 bar. The H_2/CO_2 selectivity of the substrate which supported the thin MXene membranes was 4.6, which is comparable with previous work.
